# Association between type 2 diabetes and amyotrophic lateral sclerosis

**DOI:** 10.1038/s41598-022-06463-6

**Published:** 2022-02-15

**Authors:** Linjing Zhang, Lu Tang, Tao Huang, Dongsheng Fan

**Affiliations:** 1 Department of Neurology, Peking University Third Hospital, 49 North Garden Road, Haidian District, Beijing, 100191 China; 2 Department of Epidemiology and Biostatistics, School of Public Health, Peking University, Beijing, China; 3 Key Laboratory of Molecular Cardiovascular Sciences (Peking University), Ministry of Education, Beijing, China; 4 Beijing Key Laboratory of Biomarker and Translational Research in Neurodegenerative Disorders, Beijing, China

**Keywords:** Neurology, Neurological disorders, Motor neuron disease

## Abstract

Type 2 diabetes (T2D) and amyotrophic lateral sclerosis (ALS) are associated consistently. However, it is currently unknown whether this association is causal. We aimed to estimate the unconfounded, causal association between T2D on ALS using a two-sample Mendelian randomization approach both in European and East Asian ancestry. Genetic variants strongly associated with T2D and each T2D markers were used to investigate the effect of T2D on ALS risk in European (involving 20,806 ALS cases and 59,804 controls) and East Asian (involving 1234 ALS cases and 2850 controls) ancestry. We found that the OR of ALS per 1 SD increase in T2D was estimated to be 0.96 [95% confidence interval (CI) 0.92–0.996; p = 0.03] in European populations. Similarly, all 8 SNPs were associated with T2D in East Asian ancestry, the OR of ALS per 1 SD increase in T2D was estimated to be 0.83 [95% CI 0.70–0.992; p = 0.04] in East Asian populations. Examining the intercept estimates from MR-Egger regression also leads to the same conclusion, in that horizontal pleiotropy unlikely influences the results in either population. We found that genetically predicted T2D was associated with significantly lower odds of amyotrophic lateral sclerosis both in European and East Asian populations. It is now critical to identify a clear molecular explanation for this association between T2D and ALS and to focus on its potential therapeutic implications.

## Introduction

**Figure 1:**
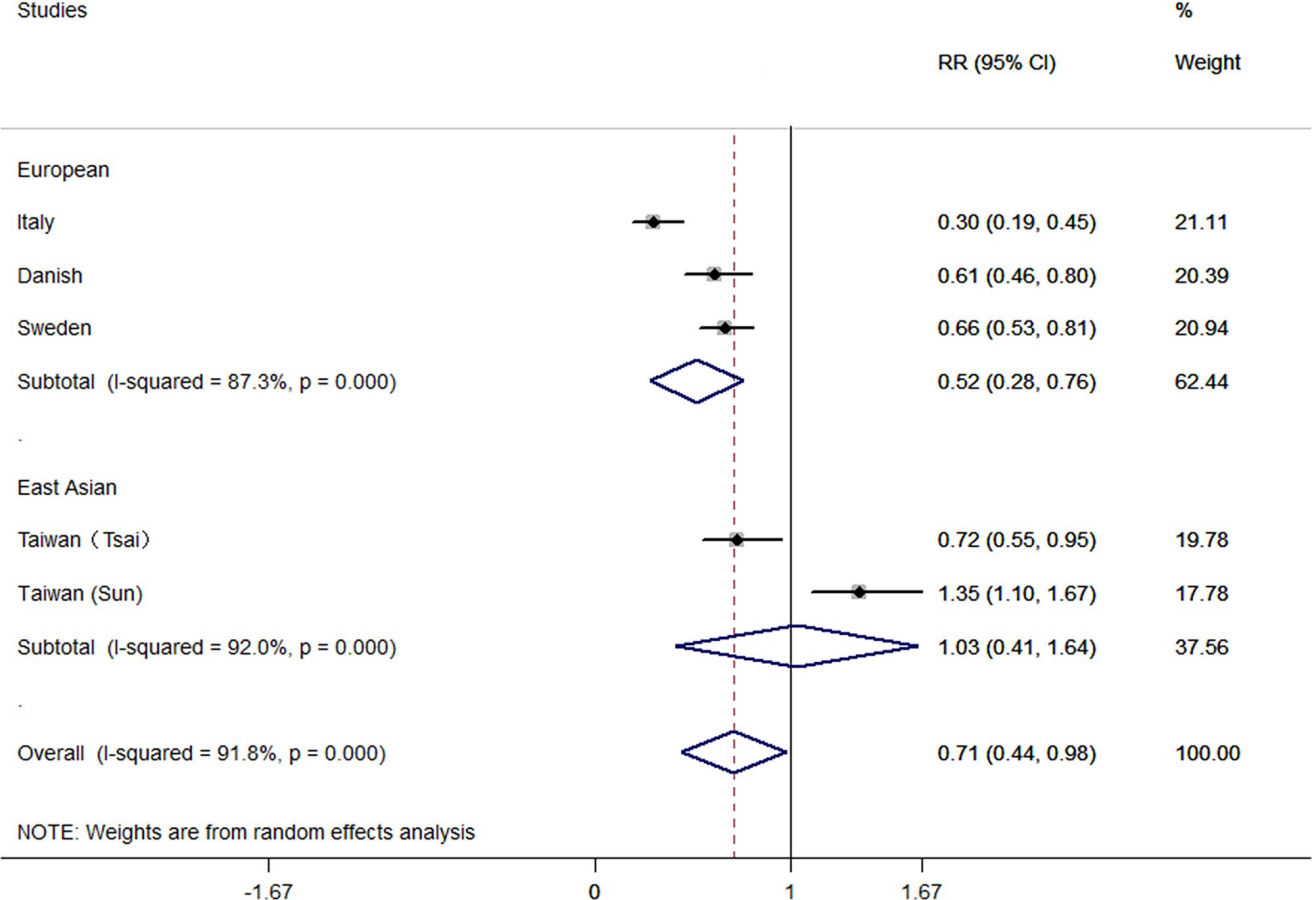
Subgroup meta-analysis of the effects on risk of ALS in patients with premorbid T2D between European populations and East Asian populations. *ES* effect size, *CI* confidence interval.

Recently, premorbid T2D has been proposed as a identified factor for the risk of amyotrophic lateral sclerosis (ALS). Population-based cohort studies in European ancestries^1–3^ may support the protective role of diabetes in ALS (subgroup meta-analyses, odds ratio (OR) 0.52, 95% confidence interval (CI) 0.28–0.76) (Fig. 1). However, in East Asian ancestry, the association of T2D on ALS were conflicting. Population-based cohort study performed by Sun et al. in Taiwan^4^ showed a positive association between T2D and ALS with an increased risk of ALS onset (HR 1.35, 95% CI 1.10–1.67). While another population-based cohort study^5^ reported the late-onset of T2D (first T2D diagnosis was ≥ 55 years) may exert negative association with ALS (HR 0.72, 95% CI 0.55–0.95, p = 0.019). Tsai et al. attributed the difference in their results and those reported by Sun et al. may be due to the validation of ALS and the coverage of the population.

In addition, these conflicting results drawn from different regions might be attributed to the different ethnicity of the patient cohort^6^. Observational studies are prone to potential confounding (such as obesity, hyperlipidemia and T2D are often co-morbid) and reverse causation and various biases and have generated findings that have suggested to be unreliable indicators of the causal effects of modifiable exposures on disease outcomes.

Additionally, there were no data in any of these studies on the duration and severity of diabetes, which might limit the assessment of dose–response relationships, one of the key features of causality when it is assessed in observational studies. Therefore, whether T2D is causally associated with the risk of ALS remains largely unknown. Mendelian randomization (MR) is one of the emerging approaches to strengthen causal inference using the instrumental variable (IV) method^7^. This approaches were designed to minimize the identified confounding and avoid reverse causation, and to some extent Mendelian randomization can be thought of as a “natural” randomized controlled trial. MR is a potentially robust method that can support the resolution of observational correlations into causal relationships, which is an elusive problem at the heart of biological understanding, pharmaceutical development, prevention of disease and medical practice.

Here, we exploited the two-sample MR design to examine the causal effects of T2D on ALS in both European and East Asian populations. We hypothesize that T2D was causally inverse associated with the risk of ALS.

## Materials and methods

### European ancestry

For exposure, we utilized 139 near-independent variants (UKB genotypes: clumping r2 threshold = 0.01 and window size = 1 Mb) of T2D at genome-wide significance (p < 5 × 10^−8^) as instrumental variables for MR analysis, including 62,892 European ancestry cases and 596,424 European ancestry controls^8^). These SNPs explained 19.6% (s.e. = 0.011) of the variability in T2D^8^. F‐statistics was 1057, larger than the conventional value of 10, indicating that the instruments had strong potential to predict T2D^9^. In addition, 40 SNPs of fasting glucose^10^, 19 SNPs of fasting insulin^10^, 9 SNPs of 2 h glucose^10^ at genome-wide significance (p < 5 × 10^−8^) were identified from up to 133,010 (fasting glucose), 108,557 (fasting insulin) and 42,854 (2 h Glu) non-diabetic individuals of European ancestry, including individuals from the previous meta-analyses^11,12^. Fasting proinsulin^13^, and HbA1C^14^ at genome-wide significance (p < 5 × 10^−8^) were identified from the study of large-scale genome-wide association analyses. Summary statistics for the genetic variants associated with T2D and related traits used in the MR analyses are provided in Table 1.

**Table 1:** Characteristics of instrumental variables used in the Mendelian randomisation analyses in European ancestry.

Exposure	Published genome-wide association study of the T2D etc			Present Mendelian randomisation analysis		
	References	Maximum sample size	No. of independent genome-wide significant SNPs	No. of SNPs inclueded in the analysis	Proxies used (r2 > 0.9)	Excluded (no proxy at r2 > 0.9)
T2D	Xue et al.^8^	721,534	139	139		
2 h glucose	Scott et al.^10^	133,010	7	7		
Fasting glucose	Scott et al.^10^	133,010	40	40		
Fasting insulin	Scott et al.^10^	133,010	15	15		
Fasting proinsulin	Strawbridge et al.^13^	16,378	10	10		
HbA1C	Soranzo et al.^14^	46,368	11	11		

*SNPs* single nucleotide polymorphisms, *T2D* type 2 diabetes, *HbA1C* hemoglobin A1c.

For each aforementioned exposure-associated SNP, we retrieved GWAS summary statistics from the ALS Variant Server (AVS), the largest GWAS to date, which includes 20,806 ALS cases and 59,804 controls in populations of European ancestry^15^, to achieve the number of SNPs along with their beta effect and standard errors. In all, the number of IVs for T2D, 2 h glucose, fasting glucose, fasting insulin, fasting proinsulin, and HbA1C were 139, 7, 40, 15, 10, 11, respectively, and full details of the selected SNPs, including their beta effect and standard errors in each GWAS summary statistics, are provided in Table S1.

### East Asian ancestry

For exposure, as instrumental variables for the MR analysis, we utilized 8 SNPs associated with T2D at genome-wide significance (p < 5 × 10^−8^) identified from a meta-analysis of GWAS data involving 16,005 individuals with T2D and 16,969 healthy control individuals of East Asian ancestry^16^. The heritability of the IVs for T2D was not provided in the original paper, according to previous related studies, it was estimated about 15%^16^. F-statistics of IVs was 839^9^. In addition, SNPs that have been significantly associated with glucose^17^, glycated hemoglobin^18^, body mass index (BMI)^19^, and WCadjBMI (waist circumference)^20^ at genome-wide significance (p < 5 × 10^−8^) were derived from a GWAS meta-analysis. Characteristics of these selected instrumental variables for East Asian ancestry used in the MR analyses are provided in Table 2.

**Table 2:** Characteristics of instrumental variables used in the Mendelian randomization analyses in East Asian ancestry.

Exposure	Published genome-wide association study of the T2D etc			Present Mendelian randomization analysis		
	References	Maximum sample size	No. of independent genome-wide significant SNPs	No. of SNPs included in the analysis	Proxies used (r2 > 0.9)	Excluded (no proxy at r2 > 0.9)
T2D	Li et al.^16^	32,974	10	8		2
Glucose	Hwang et al.^17^	24,740	14	14		
Glycated hemoglobin	Chen et al.^18^	~30,000	12	11		1
BMI	Wen et al.^19^	27,715	13	12		1
WCadjBMI	Wen et al.^20^	16,378	4	4		

*SNPs* single nucleotide polymorphisms, *T2D* type 2 diabetes, *BMI* body mass index.

For each aforementioned exposure-associated SNP, we extracted its corresponding SNP number along with their beta effect and standard errors from GWAS summary statistics for ALS involving 1234 ALS cases and 2850 controls of Chinese Han ancestry^21^. In all, the number of IVs for T2D, glucose, glycated hemoglobin, BMI and WCadjBMI were 8, 14, 11, 12, 4, respectively, and full details of the selected SNPs, including their beta effect and standard errors in each GWAS summary statistic, are provided in Table S2.

## Mendelian randomization analysis

**Figure 2:**
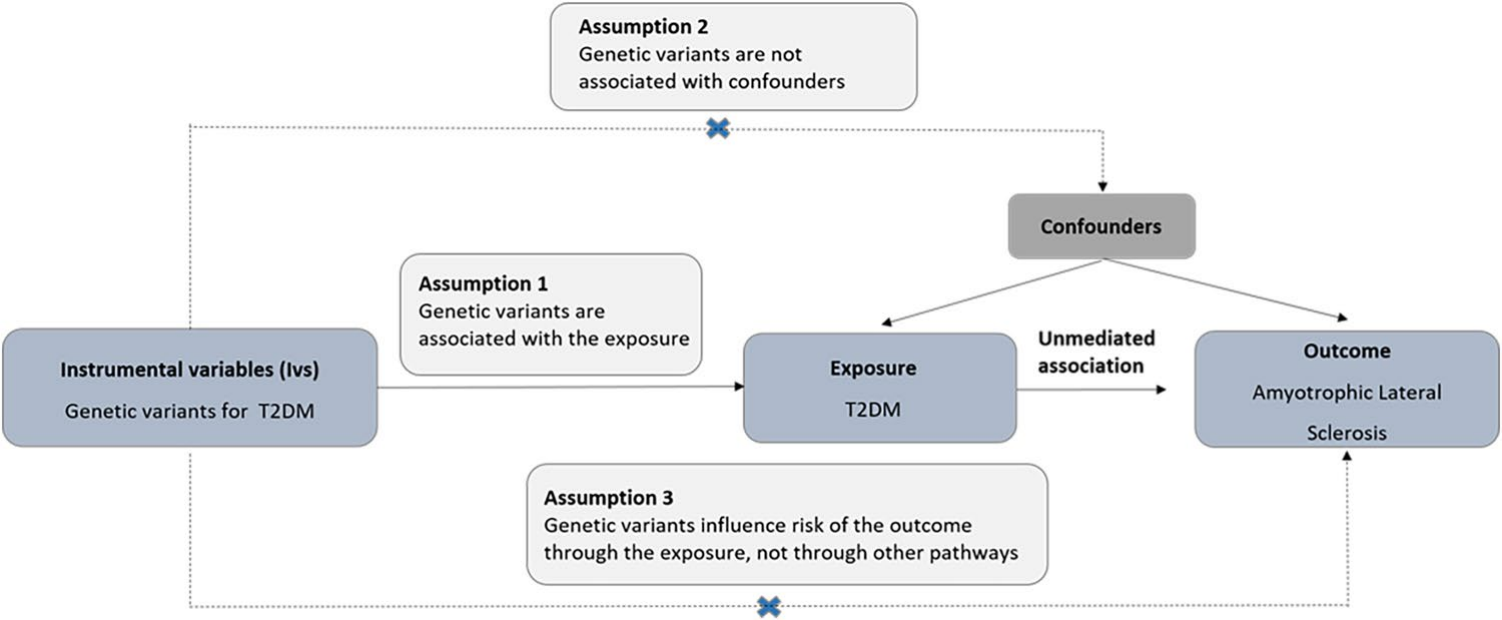
Assumptions of a Mendelian randomization analysis between T2D and risk of amyotrophic lateral. Broken lines represent potential pleiotropic or direct causal effects between variables that would violate Mendelian randomization assumptions. *T2D* type 2 diabetes mellitus.

The hypothesis^7^ and analysis was descripted in our previous study^22^ (Fig. 2). For each IV described above, an IV ratio estimate was calculated by dividing the effect size estimate for the association of the variant with the risk of ALS by the corresponding estimate for the association of the variant with the T2D. Then, we performed the conventional fixed effect inverse variance weighted (IVW) method^23^, simple median, weighted median and MR-Egger regression methods^24^ for the ratio estimates. The results are presented as ORs (95% CI) per genetically predicted 1 SD increase in each trait. No adjustment was made for multiple statistical testing, as this study was based on the primary hypothesis that T2D was associated with ALS. The statistical analyses were conducted using R version 3.5.3 (R Project for Statistical Computing) and Stata version 11.2 (Stata Corp, College Station, TX).

All human research was conducted according to the Declaration of Helsinki. Ethical approval was obtained from the review boards of Peking University Third Hospital.

### Ethics approval

Informed consent was obtained from all participants of the contributing studies, which had received ethical approval from their respective institutional review boards.

## Results

### Causal effects of T2D on ALS in the European population

**Figure 3:**
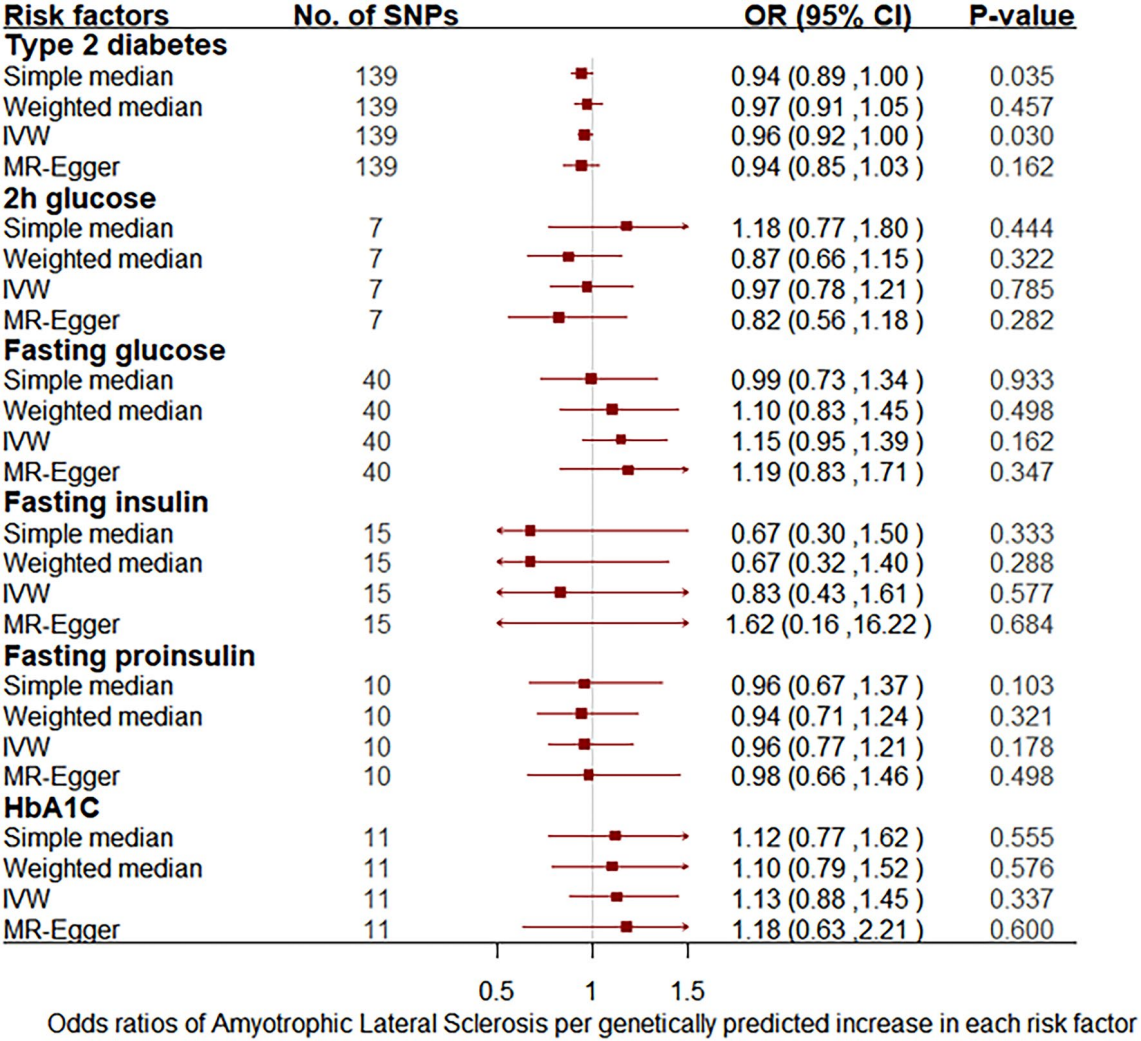
Odds ratio for association between genetically predicted T2D and related traits and ALS in European populations. Squares represent the odds ratio of amyotrophic lateral sclerosis per genetically predicted 1 SD increase in T2D; horizontal lines represent 95% confidence intervals (CIs). *SNPs* single nucleotide polymorphisms, *OR* odds ratio, *IVW* inverse variance weighted, *HbA1C* hemoglobin A1C.

Using MR analysis, we found that genetically predicted T2D was associated with significantly lower odds of ALS in European populations. Specifically, the odds ratio (OR) of ALS per 1 standard deviation (SD) increase in T2D is estimated to be 0.96 [95% CI 0.92–0.996; p = 0.03], suggesting that higher predisposition of T2D is causally associated with a decreased risk of ALS (Fig. 3). In addition, the associations were consistent in sensitivity analyses that used simple medians but with less precision (Fig. 3, Table S3). This association was driven by rsid (rs) 1,758,632 (0.31, 0.15–0.62; p = 0.0008), rs12681990 (0.53, 0.29–0.97; p = 0.04) and rs72802358 (0.65, 0.44–0.95; p = 0.03) (Fig. S1). In the MR-Egger analysis, there was no evidence of directional pleiotropy (intercept 0.002 ± 0.003; p = 0.615), however, a low I2 test may mean MR Egger is underpowered to rule out a pleiotropic effect through inspecting the intercept.

We did not observe a significant association between 2 h glucose, fasting glucose, fasting insulin, fasting proinsulin, and HbA1C and the risk of ALS in European populations (Fig. 3, Table S3). Specifically, the OR of ALS per 1 SD increase in 2 h glucose, fasting glucose, fasting insulin, fasting proinsulin, and HbA1C were estimated to be 0.97 [95% CI 0.78–1.21; p = 0.785], 1.15 [95% CI 0.95–1.39; p = 0.162], 0.83 [95% CI 0.43–1.61; p = 0.577], 0.96 [95% CI 0.77–1.21; p = 0.178], and 1.13 [95% CI 0.88–1.45; p = 0.337] (Fig. 3).

### Causal effects of T2D on ALS in the East Asian population

**Figure 4:**
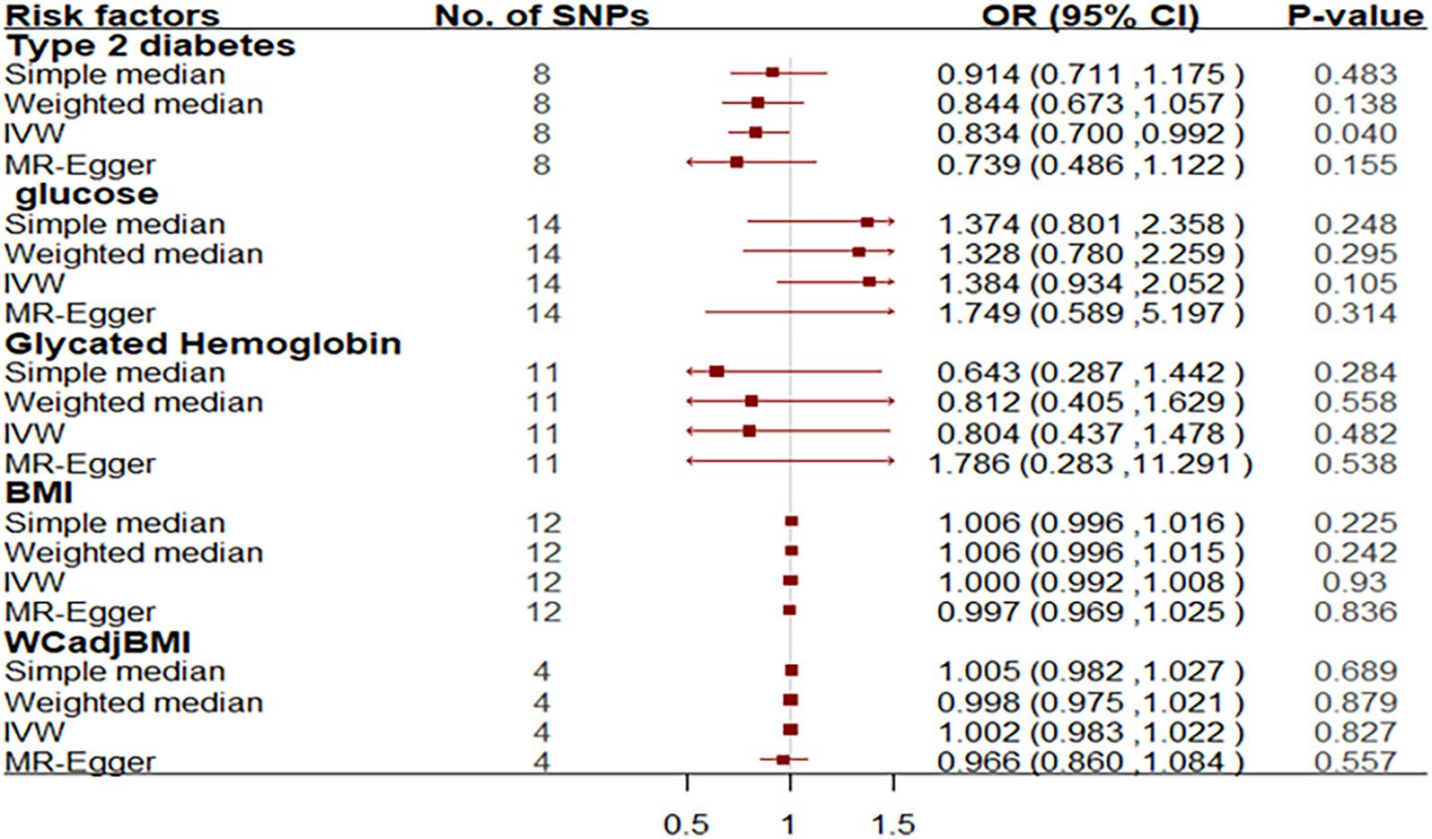
Odds ratio for association between genetically predicted T2D and related traits and ALS in East Asian populations. Squares represent the odds ratio of amyotrophic lateral sclerosis per genetically predicted 1 SD increase in T2D; horizontal lines represent 95% confidence intervals (CIs). *SNPs* single nucleotide polymorphisms, *OR* odds ratio, *IVW* inverse variance weighted, *BMI* body mass index.

Similarly, genetically predicted T2D was associated with significantly lower odds of ALS in East Asian populations. Specifically, the OR of ALS per 1 SD increase in T2D was estimated to be 0.83 [95% CI 0.70–0.992; p = 0.040], suggesting that higher predisposition of T2D is causally associated with a decreased risk of ALS (Fig. 4). This association was driven by rs4430796 (0.52, 0.27–1.00; p = 0.05) (Fig. S2). The MR-Egger regression intercept was estimated to be 0.027 (95% CI − 0.016 to 0.07; *p* = 0.533), leading to horizontal pleiotropy, which is unlikely to influence results in the East Asian population (Table S4).

In contrast, the OR of ALS per 1 SD increase in glucose was 1.38 (95% CI 0.93–2.05; p = 0.105), and the OR of ALS per 1 SD increase in glycated hemoglobin was 0.80 (95% CI 0.44–1.48; p = 0.482); both are not statistically significant using the IVW analyses method. Neither BMI nor WCadjBMI was found to be causally associated with the risk of ALS. Specifically, the OR of ALS per 1 SD increase in BMI was estimated to be 1.00 [95% CI 0.99–1.00; p = 0.930], and the WCadjBMI was estimated to be 1.00 [95% CI 0.98–1.020; p = 0.827] (Fig. 4).

## Discussion

This comprehensive MR study provide important evidence supporting genetically predicted T2D may decrease the risk of ALS in both European and East Asian populations. The sensitivity analysis also leads to the same conclusion that horizontal pleiotropy unlikely influences results in either population.

The pathophysiological mechanisms underlying the relationship between T2D and ALS remain largely unknown because we do not have sufficient evidence. ALS is a rare disease, and its pathogenesis is very difficult to study with retrospective data from different populations^25^. In this MR study, none of other metrics were found to be causally associated with the risk of ALS, we first speculated that, the effects of these metrics (glucose, glycated hemoglobin, BMI, WCadjBMI) on risk of ALS may be not strong enough to provide genetic evidence so their effect on ALS could not be determined using MR analysis. An alternative explanation is that combined with the results that type 1 diabetes (a disorder characterized by absolute lack of insulin and abnormally high blood sugar levels) was associated with an increased risk of ALS^1,2^, it is indicative that the protective role of T2D on ALS more likely comes from other unknown signaling pathways rather than the currently proposed pathophysiology occurring in the body. Although the effects in this study were small, it added genetic evidence to further understand this topic. Thus, it should be a great priority for the ALS research community to unravel the molecular underpinnings of the protective effect of T2D in ALS. This holds tremendous therapeutic promise and accelerates translates this finding into desperately needed treatments for ALS.

Limitations of our study include that the samples used to define the instrumental variable for T2D were same with the samples from the ALS consortium. Also, completely ruling out pleiotropy (assumption 2) or an alternative direct causal pathway (assumption 3) is challenge for all MR analyses, for there were probably some unknown confounders could influence the ALS^26^. Genetic variants of T2D in European and East Asian ancestry that were associated with other traits were provided in Table S5. Nevertheless, our study assumed a linear relationship between T2D and ALS and did not investigate nonlinear effects of T2D. Furthermore, to examine the possibility that our results were driven by reverse causality, we tried to perform IVW analysis in the reverse direction to examine the causal effects of ALS on T2D, but the power of IVs for ALS were too small (no more than 4 SNPs could be calculated the ratio estimate) to perform bidirectional MR analyze. Finally, the associations between T2D and ALS observed from previous studies are varied in young and aged populations^6^. Hence, bias from population stratification is deemed likely^27^.

## Supplementary Information

Supplementary Information.

## Data Availability

The corresponding author has full access to all data and material and can provide availability if needed.

## References

[1] Kioumourtzoglou MA, Rotem RS, Seals RM, Gredal O, Hansen J, Weisskopf MG (2015). Diabetes mellitus, obesity, and diagnosis of amyotrophic lateral sclerosis: A population-based study. JAMA Neurol..

[2] Mariosa D, Kamel F, Bellocco R, Ye W, Fang F (2015). Association between diabetes and amyotrophic lateral sclerosis in Sweden. Eur. J. Neurol..

[3] D'Ovidio F, d'Errico A, Carna P, Calvo A, Costa G, Chio A (2018). The role of pre-morbid diabetes on developing amyotrophic lateral sclerosis. Eur. J. Neurol..

[4] Sun Y, Lu CJ, Chen RC, Hou WH, Li CY (2015). Risk of amyotrophic lateral sclerosis in patients with diabetes: A Nationwide Population-Based Cohort Study. J. Epidemiol..

[5] Tsai CP, Lee JK, Lee CT (2019). Type II diabetes mellitus and the incidence of amyotrophic lateral sclerosis. J. Neurol..

[6] Logroscino G (2015). Motor neuron disease: Are diabetes and amyotrophic lateral sclerosis related?. Nat. Rev. Neurol..

[7] Davey Smith G, Hemani G (2014). Mendelian randomization: Genetic anchors for causal inference in epidemiological studies. Hum. Mol. Genet..

[8] Xue A, Wu Y, Zhu Z, Zhang F, Kemper KE, Zheng Z, Yengo L, Lloyd-Jones LR, Sidorenko J, Wu Y (2018). Genome-wide association analyses identify 143 risk variants and putative regulatory mechanisms for type 2 diabetes. Nat. Commun..

[9] Pierce BL, Ahsan H, Vanderweele TJ (2011). Power and instrument strength requirements for Mendelian randomization studies using multiple genetic variants. Int. J. Epidemiol..

[10] Scott RA, Lagou V, Welch RP, Wheeler E, Montasser ME, Luan J, Magi R, Strawbridge RJ, Rehnberg E, Gustafsson S (2012). Large-scale association analyses identify new loci influencing glycemic traits and provide insight into the underlying biological pathways. Nat. Genet..

[11] Dupuis J, Langenberg C, Prokopenko I, Saxena R, Soranzo N, Jackson AU, Wheeler E, Glazer NL, Bouatia-Naji N, Gloyn AL (2010). New genetic loci implicated in fasting glucose homeostasis and their impact on type 2 diabetes risk. Nat. Genet..

[12] Saxena R, Hivert MF, Langenberg C, Tanaka T, Pankow JS, Vollenweider P, Lyssenko V, Bouatia-Naji N, Dupuis J, Jackson AU (2010). Genetic variation in GIPR influences the glucose and insulin responses to an oral glucose challenge. Nat. Genet..

[13] Strawbridge RJ, Dupuis J, Prokopenko I, Barker A, Ahlqvist E, Rybin D, Petrie JR, Travers ME, Bouatia-Naji N, Dimas AS (2011). Genome-wide association identifies nine common variants associated with fasting proinsulin levels and provides new insights into the pathophysiology of type 2 diabetes. Diabetes.

[14] Soranzo N, Sanna S, Wheeler E, Gieger C, Radke D, Dupuis J, Bouatia-Naji N, Langenberg C, Prokopenko I, Stolerman E (2010). Common variants at 10 genomic loci influence hemoglobin A(1)(C) levels via glycemic and nonglycemic pathways. Diabetes.

[15] Nicolas A, Kenna KP, Renton AE, Ticozzi N, Faghri F, Chia R, Dominov JA, Kenna BJ, Nalls MA, Keagle P (2018). Genome-wide analyses identify KIF5A as a novel ALS gene. Neuron.

[16] Li H, Gan W, Lu L, Dong X, Han X, Hu C, Yang Z, Sun L, Bao W, Li P (2013). A genome-wide association study identifies GRK5 and RASGRP1 as type 2 diabetes loci in Chinese Hans. Diabetes.

[17] Hwang JY (2015). Genome-wide association meta-analysis identifies novel variants associated with fasting plasma glucose in East Asians. Diabetes.

[18] Chen P, Takeuchi F, Lee JY, Li H, Wu JY, Liang J, Long J, Tabara Y, Goodarzi MO, Pereira MA (2014). Multiple nonglycemic genomic loci are newly associated with blood level of glycated hemoglobin in East Asians. Diabetes.

[19] Wen W, Cho YS, Zheng W, Dorajoo R, Kato N, Qi L, Chen CH, Delahanty RJ, Okada Y, Tabara Y (2012). Meta-analysis identifies common variants associated with body mass index in east Asians. Nat. Genet..

[20] Wen W, Kato N, Hwang JY, Guo X, Tabara Y, Li H, Dorajoo R, Yang X, Tsai FJ, Li S (2016). Genome-wide association studies in East Asians identify new loci for waist-hip ratio and waist circumference. Sci. Rep..

[21] Benyamin B, He J, Zhao Q, Gratten J, Garton F, Leo PJ, Liu Z, Mangelsdorf M, Al-Chalabi A, Anderson L (2017). Cross-ethnic meta-analysis identifies association of the GPX3-TNIP1 locus with amyotrophic lateral sclerosis. Nat. Commun..

[22] Zhang L, Tang L, Huang T, Fan D (2020). Life course adiposity and amyotrophic lateral sclerosis: A Mendelian randomization study. Ann. Neurol..

[23] Burgess S, Bowden J, Fall T, Ingelsson E, Thompson SG (2017). Sensitivity analyses for robust causal inference from Mendelian randomization analyses with multiple genetic variants. Epidemiology.

[24] Burgess S, Thompson SG (2017). Interpreting findings from Mendelian randomization using the MR-Egger method. Eur. J. Epidemiol..

[25] Jawaid A, Abid A, Schulz PE (2018). Diabetes mellitus and amyotrophic lateral sclerosis: Time to bridge the gap between the bench and the bedside. Eur. J. Neurol..

[26] Burgess S, Butterworth AS, Thompson JR (2016). Beyond Mendelian randomization: How to interpret evidence of shared genetic predictors. J. Clin. Epidemiol..

[27] Smith GD, Lawlor DA, Harbord R, Timpson N, Day I, Ebrahim S (2007). Clustered environments and randomized genes: A fundamental distinction between conventional and genetic epidemiology. PLoS Med..

