# Supplementary material for: Association between type 2 diabetes and amyotrophic lateral sclerosis
Source: Sci Rep. 2022 Feb 15;12:2544. doi: 10.1038/s41598-022-06463-6 (PMC8847454; doi:10.1038/s41598-022-06463-6)
Supplement: Supplementary file 1 — Supplementary Information. [file 41598_2022_6463_MOESM1_ESM.docx]

**Supplemental material**

**Figure S1.** Association between genetically predicted T2D and ALS in European populations.

**Figure S2.** Association between genetically predicted T2D and ALS in East Asian populations.

**Table S1.** Summary statistics for the genetic variants associated with T2D and related traits investigated for an association with ALS in European ancestry.

**Table S2.** Summary statistics for the genetic variants associated with T2D and related traits investigated for an association with ALS in East Asian ancestry.

**Table S3.** Results of four MR methods of MR analysis for T2D and related traits in European ancestry.

**Table S4.** Results of four MR methods of MR analysis for T2D and related traits in East Asian ancestry.

**Table S5.** Genetic variants of T2D in European and East Asian ancestry that were associated with other traits.

**Figure S1.** Association between genetically predicted T2D and ALS in European populations.


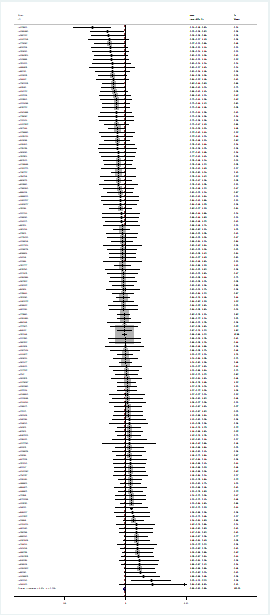


Note: Squares represent the odds ratio of ALS per genetically predicted 1 SD increase in T2D; horizontal lines represent 95% confidence intervals (CIs); diamond represent the overall odds ratio with its 95% CI. SNPs = single nucleotide polymorphisms.

**Figure S2.** Association between genetically predicted T2D and ALS in East Asian populations.

**
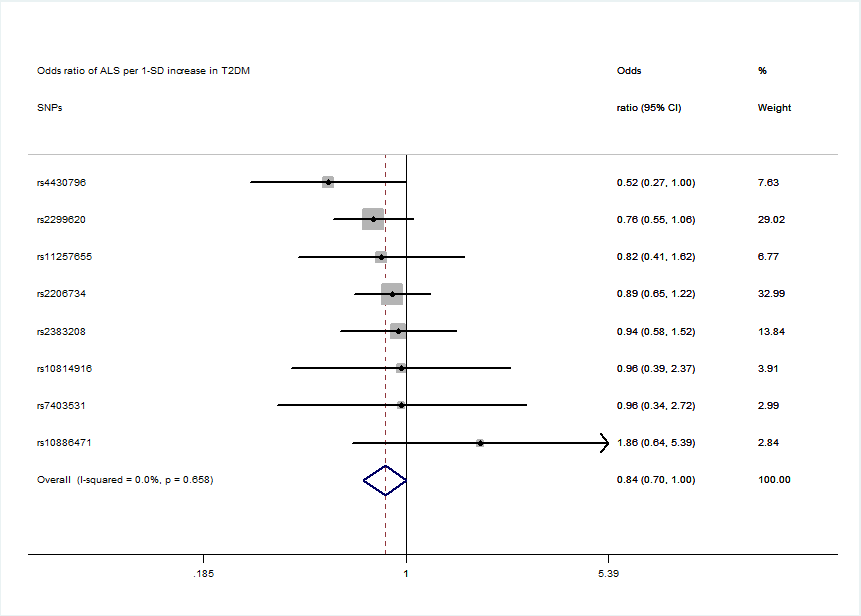
**

Note: Squares represent the odds ratio of ALS per genetically predicted 1 SD increase in T2D; horizontal lines represent 95% confidence intervals (CIs); diamond represent the overall odds ratio with its 95% CI. SNPs = single nucleotide polymorphisms.

| **Table S1.** Summary statistics for the genetic variants associated with T2D and related traits investigated for an association with ALS in European ancestry. | | | | | | | | | | | |
| --- | --- | --- | --- | --- | --- | --- | --- | --- | --- | --- | --- |
| **Risk factors** | **SNP** | **Chr** | **Nearest gene** | **EA** | **Risk Factor** | | | **NGWAS** | **ALS** | | |
|  |  |  |  |  | **Beta** | **SE** | **P value** |  | **Beta** | **SE** | **P value** |
| Type 2 diabetes | rs10077431 | 5 | *YTHDC2* | A | -0.049 | 0.009 | 4.76E-08 | 655808 | 0.0049 | 0.0173 | 0.776 |
| Type 2 diabetes | rs1009358 | 2 | *PURG* | C | -0.055 | 0.008 | 9.81E-12 | 582408 | 0.0247 | 0.0139 | 0.07568 |
| Type 2 diabetes | rs10100265 | 8 | *CEP68* | A | 0.049 | 0.008 | 6.29E-10 | 590089 | 0.0086 | 0.0139 | 0.5344 |
| Type 2 diabetes | rs10114341 | 9 | *PINX1* | C | -0.041 | 0.007 | 1.15E-08 | 685351 | 0.0034 | 0.0136 | 0.8053 |
| Type 2 diabetes | rs10169613 | 2 | *LOC107987099 (PTPDC1)* | T | -0.043 | 0.008 | 3.57E-08 | 577559 | 0.0031 | 0.0139 | 0.8206 |
| Type 2 diabetes | rs10401969 | 19 | *BCL2L11* | C | 0.092 | 0.013 | 4.13E-12 | 700035 | 0.0295 | 0.0267 | 0.2686 |
| Type 2 diabetes | rs1042725 | 12 | *SUGP1* | T | 0.056 | 0.007 | 3.37E-15 | 695009 | 0.0218 | 0.0137 | 0.1116 |
| Type 2 diabetes | rs1050226 | 6 | *RREB1* | G | -0.049 | 0.007 | 3.34E-11 | 662713 | 0.0179 | 0.0138 | 0.1968 |
| Type 2 diabetes | rs1061813 | 5 | *ANKH* | G | 0.043 | 0.007 | 3.37E-09 | 660981 | 0.0119 | 0.0139 | 0.3931 |
| Type 2 diabetes | rs1063355 | 6 | *CDKN2B-AS1/CDKN2B* | T | -0.071 | 0.008 | 3.72E-19 | 585822 | 0.0053 | 0.0139 | 0.7025 |
| Type 2 diabetes | rs10757283 | 9 | *HLA-DQB1* | T | 0.029 | 0.007 | 9.85E-05 | 647851 | 0.0042 | 0.014 | 0.7637 |
| Type 2 diabetes | rs10811661 | 9 | *CDKN2B-AS1* | C | -0.157 | 0.01 | 4.13E-58 | 635360 | 0.007 | 0.019 | 0.7105 |
| Type 2 diabetes | rs10830963 | 11 | *MTNR1B* | G | 0.091 | 0.008 | 5.85E-30 | 685037 | 0.0061 | 0.0159 | 0.7002 |
| Type 2 diabetes | rs10842994 | 12 | *LOC105369709 (KLHL42)* | T | -0.076 | 0.009 | 1.02E-16 | 668415 | 0.0058 | 0.0172 | 0.736 |
| Type 2 diabetes | rs10974438 | 9 | *GLIS3* | C | 0.059 | 0.008 | 3.01E-15 | 683222 | 0.0237 | 0.0145 | 0.1016 |
| Type 2 diabetes | rs11098676 | 4 | *ITPR2* | T | -0.054 | 0.01 | 2.03E-08 | 568106 | 0.0334 | 0.0162 | 0.03956 |
| Type 2 diabetes | rs11107116 | 12 | *NUDT6* | T | 0.047 | 0.009 | 3.75E-08 | 707016 | 0.0276 | 0.0167 | 0.09967 |
| Type 2 diabetes | rs1111875 | 10 | *SOCS2* | T | -0.095 | 0.007 | 3.62E-39 | 699127 | 0.0001 | 0.0138 | 0.9956 |
| Type 2 diabetes | rs11257655 | 10 | *CDC123* | T | 0.074 | 0.009 | 1.97E-17 | 705380 | 0.0229 | 0.0168 | 0.1733 |
| Type 2 diabetes | rs1127655 | 1 | *PTGFRN* | C | 0.044 | 0.008 | 2.47E-08 | 563183 | 0.0125 | 0.0136 | 0.3578 |
| Type 2 diabetes | rs11591741 | 10 | *CHUK* | C | -0.048 | 0.008 | 1.23E-09 | 584062 | 0.0135 | 0.0137 | 0.3253 |
| Type 2 diabetes | rs11651755 | 17 | *HNF1B* | C | 0.074 | 0.008 | 8.98E-22 | 591312 | 0.0048 | 0.0138 | 0.7257 |
| Type 2 diabetes | rs11708067 | 3 | *ADCY5* | G | -0.097 | 0.009 | 5.93E-29 | 650949 | 0.0053 | 0.0166 | 0.7501 |
| Type 2 diabetes | rs11925227 | 3 | *LOC157273(TNKS)* | A | -0.053 | 0.01 | 2.25E-08 | 647813 | 0.0093 | 0.0184 | 0.6139 |
| Type 2 diabetes | rs11926707 | 3 | *TNIK* | T | -0.046 | 0.008 | 1.69E-08 | 556022 | 0.008 | 0.0143 | 0.5771 |
| Type 2 diabetes | rs12088739 | 1 | *PTH1R* | G | -0.088 | 0.013 | 9.79E-12 | 633660 | 0.0088 | 0.0239 | 0.7135 |
| Type 2 diabetes | rs12299509 | 12 | *MIR4421* | G | 0.047 | 0.007 | 2.09E-10 | 658536 | 0.0069 | 0.0146 | 0.6362 |
| Type 2 diabetes | rs12617659 | 2 | *CCND2* | T | -0.069 | 0.01 | 2.83E-11 | 657424 | 0.0308 | 0.0202 | 0.1279 |
| Type 2 diabetes | rs12681990 | 8 | *LOC105373585 (GLI2)* | C | 0.063 | 0.01 | 3.62E-11 | 693991 | 0.0402 | 0.0186 | 0.03038 |
| Type 2 diabetes | rs12910825 | 15 | *KCNU1* | G | 0.052 | 0.007 | 2.16E-12 | 693769 | 0.0264 | 0.014 | 0.05976 |
| Type 2 diabetes | rs12945601 | 17 | *PRC1* | T | 0.048 | 0.008 | 1.72E-09 | 577125 | 0.0243 | 0.0139 | 0.08019 |
| Type 2 diabetes | rs12970134 | 18 | *RAI1* | A | 0.056 | 0.008 | 5.31E-12 | 702309 | 0.0006 | 0.0154 | 0.9713 |
| Type 2 diabetes | rs13234269 | 7 | *MC4R* | A | -0.058 | 0.008 | 6.98E-14 | 575869 | 0.0191 | 0.0141 | 0.175 |
| Type 2 diabetes | rs13239186 | 7 | *LOC105375508 (KLF14)* | T | 0.054 | 0.009 | 2.7E-10 | 574968 | 0.0041 | 0.0152 | 0.7872 |
| Type 2 diabetes | rs13389219 | 2 | *CTTNBP2* | T | -0.072 | 0.007 | 2.11E-22 | 669517 | 0.008 | 0.0142 | 0.5713 |
| Type 2 diabetes | rs1359790 | 13 | *COBLL1* | A | -0.08 | 0.008 | 2.8E-23 | 669042 | 0.0165 | 0.0154 | 0.2849 |
| Type 2 diabetes | rs1496653 | 3 | *LOC105370275 (SPRY2)* | G | -0.077 | 0.009 | 2.57E-18 | 694395 | 0.0141 | 0.017 | 0.4065 |
| Type 2 diabetes | rs1552224 | 11 | *HMGA2* | C | -0.103 | 0.01 | 8.64E-25 | 658127 | -0.007 | 0.019 | 0.714 |
| Type 2 diabetes | rs16988333 | 22 | *UBE2E2* | G | -0.075 | 0.013 | 9.17E-09 | 630165 | 0.0139 | 0.0253 | 0.581 |
| Type 2 diabetes | rs17086692 | 4 | *ARAP1* | T | -0.047 | 0.008 | 2.48E-08 | 576758 | 0.0137 | 0.015 | 0.3609 |
| Type 2 diabetes | rs17168486 | 7 | *HORMAD2* | T | 0.074 | 0.009 | 2.18E-15 | 690807 | 0.0187 | 0.0184 | 0.3097 |
| Type 2 diabetes | rs17405722 | 17 | *SPATA18* | A | 0.087 | 0.015 | 2.28E-09 | 598412 | 0.0216 | 0.0272 | 0.4277 |
| Type 2 diabetes | rs17411031 | 8 | *DGKB* | G | -0.045 | 0.008 | 3.04E-08 | 690781 | 0.0167 | 0.0153 | 0.2744 |
| Type 2 diabetes | rs1758632 | 9 | *THADA* | C | -0.049 | 0.008 | 1.36E-09 | 568533 | 0.0571 | 0.0142 | 5.81E-05 |
| Type 2 diabetes | rs17791513 | 9 | *STAT3* | G | -0.103 | 0.015 | 4.61E-12 | 702314 | 0.0119 | 0.0286 | 0.6781 |
| Type 2 diabetes | rs1801214 | 4 | *LPL* | C | -0.09 | 0.007 | 5.52E-34 | 666002 | 0.011 | 0.0139 | 0.4285 |
| Type 2 diabetes | rs1899951 | 3 | *UBAP2* | T | -0.112 | 0.011 | 1.64E-24 | 681866 | 0.0203 | 0.021 | 0.3339 |
| Type 2 diabetes | rs2042073 | 8 | *TACO1* | A | 0.047 | 0.008 | 3.01E-09 | 587074 | 0.0062 | 0.0139 | 0.657 |
| Type 2 diabetes | rs2058913 | 15 | *LOC101927450 (TLE1)* | A | 0.049 | 0.008 | 3.26E-10 | 585657 | 0.0162 | 0.0137 | 0.2375 |
| Type 2 diabetes | rs2071479 | 6 | *WFS1* | T | 0.147 | 0.023 | 6.62E-11 | 644254 | 0.0116 | 0.041 | 0.7779 |
| Type 2 diabetes | rs2129869 | 12 | *PPARG* | T | 0.054 | 0.009 | 3.92E-09 | 603370 | 0.0268 | 0.0168 | 0.1118 |
| Type 2 diabetes | rs2237892 | 11 | *HLA-DOB* | T | -0.096 | 0.016 | 8.75E-10 | 606440 | 0.0018 | 0.0292 | 0.951 |
| Type 2 diabetes | rs2246012 | 6 | *AGMO* | C | 0.053 | 0.009 | 2.43E-08 | 719989 | 0.0067 | 0.0189 | 0.7217 |
| Type 2 diabetes | rs2261181 | 12 | *KCNQ1* | T | 0.099 | 0.012 | 9.18E-17 | 721534 | 0.0177 | 0.0218 | 0.4172 |
| Type 2 diabetes | rs2294120 | 8 | *ARG1, MED23* | G | -0.044 | 0.008 | 1.62E-08 | 565684 | 0.0063 | 0.0138 | 0.6486 |
| Type 2 diabetes | rs2296173 | 1 | *RPSAP52* | G | 0.065 | 0.009 | 7.66E-14 | 692542 | 0.0064 | 0.0165 | 0.6969 |
| Type 2 diabetes | rs2299383 | 7 | *ATXN7* | T | 0.041 | 0.007 | 1.49E-08 | 673117 | 0.0063 | 0.0137 | 0.6481 |
| Type 2 diabetes | rs2307111 | 5 | *ZNF34* | C | -0.041 | 0.007 | 3.03E-08 | 667892 | 0.0064 | 0.0141 | 0.6521 |
| Type 2 diabetes | rs2421016 | 10 | *MACF1* | T | -0.046 | 0.007 | 1.48E-10 | 696807 | 0.0197 | 0.0136 | 0.149 |
| Type 2 diabetes | rs243019 | 2 | *RELN* | C | 0.057 | 0.007 | 2.29E-15 | 700332 | 0.0056 | 0.0137 | 0.6842 |
| Type 2 diabetes | rs2441102 | 5 | *POC5* | A | 0.026 | 0.007 | 0.000474 | 682213 | 0.0252 | 0.0142 | 0.0757 |
| Type 2 diabetes | rs244415 | 16 | *PLEKHA1* | A | -0.047 | 0.008 | 3.88E-09 | 578854 | 0.0209 | 0.0138 | 0.1297 |
| Type 2 diabetes | rs2493394 | 1 | *MIR4432HG* | G | 0.073 | 0.011 | 1.15E-10 | 715773 | 0.0092 | 0.0225 | 0.684 |
| Type 2 diabetes | rs2616132 | 10 | *NFAT5* | A | 0.046 | 0.008 | 6.58E-09 | 577292 | 0.0009 | 0.0137 | 0.9475 |
| Type 2 diabetes | rs2633310 | 10 | *NOTCH2* | T | -0.044 | 0.008 | 2.38E-08 | 570906 | 0.0403 | 0.0141 | 0.004185 |
| Type 2 diabetes | rs2796441 | 9 | *FAM241B* | A | -0.072 | 0.007 | 1.96E-22 | 676156 | 0.0049 | 0.014 | 0.7269 |
| Type 2 diabetes | rs2820426 | 1 | *CAMK2G* | A | -0.052 | 0.007 | 1.3E-12 | 690794 | 0.0059 | 0.0141 | 0.675 |
| Type 2 diabetes | rs2857605 | 6 | *LOC101927502 (TLE1)* | C | -0.067 | 0.009 | 5.9E-14 | 650284 | 0.0051 | 0.0171 | 0.7653 |
| Type 2 diabetes | rs2867125 | 2 | *LOC102723886 (LYPLAL1)* | T | -0.06 | 0.01 | 4.33E-10 | 666684 | 0.0371 | 0.0176 | 0.03504 |
| Type 2 diabetes | rs2908282 | 7 | *NFKBIL1* | A | 0.055 | 0.009 | 4.25E-09 | 679184 | 0.0147 | 0.0179 | 0.4134 |
| Type 2 diabetes | rs2925979 | 16 | *TMEM18* | T | 0.053 | 0.008 | 9.06E-12 | 686538 | 0.0026 | 0.0149 | 0.8634 |
| Type 2 diabetes | rs2972144 | 2 | *YKT6* | A | -0.091 | 0.008 | 2.55E-34 | 680134 | 0.0277 | 0.0141 | 0.04895 |
| Type 2 diabetes | rs302864 | 17 | *CMIP* | A | 0.071 | 0.013 | 2.46E-08 | 683383 | 0.0068 | 0.0247 | 0.7834 |
| Type 2 diabetes | rs340874 | 1 | *MIR5702* | T | -0.063 | 0.007 | 8.41E-18 | 668216 | 0.0081 | 0.0139 | 0.5578 |
| Type 2 diabetes | rs348330 | 1 | *TEX14* | G | 0.049 | 0.008 | 1.86E-09 | 574851 | 0.011 | 0.0145 | 0.4489 |
| Type 2 diabetes | rs3802177 | 8 | *PROX1-AS1* | A | -0.122 | 0.008 | 2.32E-52 | 638065 | 0.0016 | 0.0149 | 0.9119 |
| Type 2 diabetes | rs3887925 | 3 | *ABCB10* | C | -0.047 | 0.008 | 2.47E-09 | 567695 | 0.0007 | 0.0136 | 0.9568 |
| Type 2 diabetes | rs3936511 | 5 | *SLC30A8* | G | 0.078 | 0.009 | 3.63E-17 | 674751 | 0.0004 | 0.0179 | 0.9804 |
| Type 2 diabetes | rs4472028 | 3 | *ST6GAL1* | T | 0.045 | 0.007 | 2.08E-10 | 703637 | 0.0072 | 0.0138 | 0.6023 |
| Type 2 diabetes | rs459193 | 5 | *C5orf67* | A | -0.071 | 0.008 | 8.81E-18 | 669693 | 0.0296 | 0.0157 | 0.05863 |
| Type 2 diabetes | rs4684859 | 3 | *MBNL1* | A | 0.035 | 0.008 | 8.79E-06 | 590139 | 0.0168 | 0.014 | 0.2285 |
| Type 2 diabetes | rs4812829 | 20 | *C2CD4A* | A | 0.053 | 0.01 | 2.44E-08 | 718829 | 0.0006 | 0.0188 | 0.9761 |
| Type 2 diabetes | rs4823182 | 22 | *C5orf67* | G | 0.048 | 0.008 | 3.36E-10 | 662284 | 0.0182 | 0.0143 | 0.2047 |
| Type 2 diabetes | rs4865796 | 5 | *HNF4A* | G | -0.053 | 0.008 | 1.33E-11 | 676672 | 0.0228 | 0.0147 | 0.1218 |
| Type 2 diabetes | rs4932261 | 15 | *SAMM50* | A | 0.058 | 0.009 | 6.23E-11 | 577817 | 0.0034 | 0.0156 | 0.8271 |
| Type 2 diabetes | rs516946 | 8 | *ARL15* | T | -0.082 | 0.009 | 3.16E-22 | 665671 | 0.0237 | 0.0156 | 0.1299 |
| Type 2 diabetes | rs518394 | 9 | *TCF7L2* | C | -0.06 | 0.007 | 8.6E-17 | 684549 | -0.009 | 0.014 | 0.5204 |
| Type 2 diabetes | rs5215 | 11 | *LOC105370965 (AP3S2)* | C | 0.068 | 0.007 | 2.09E-20 | 713158 | 0.003 | 0.0141 | 0.8313 |
| Type 2 diabetes | rs576674 | 13 | *ANK1* | G | 0.065 | 0.01 | 1.79E-11 | 667507 | 0.0076 | 0.019 | 0.6877 |
| Type 2 diabetes | rs6059662 | 20 | *KCNJ11* | A | -0.045 | 0.008 | 1.51E-08 | 628187 | 0.0108 | 0.0156 | 0.4875 |
| Type 2 diabetes | rs6066138 | 20 | *KL* | A | -0.049 | 0.008 | 1.93E-09 | 648361 | 0.0247 | 0.0157 | 0.1164 |
| Type 2 diabetes | rs61953351 | 12 | *EIF2S2* | T | -0.07 | 0.009 | 1.98E-14 | 564133 | 0.0098 | 0.0156 | 0.5307 |
| Type 2 diabetes | rs622217 | 6 | *EYA2* | C | -0.049 | 0.008 | 3.13E-10 | 591420 | 0.0083 | 0.0137 | 0.545 |
| Type 2 diabetes | rs6494307 | 15 | *OASL* | G | -0.044 | 0.008 | 1.67E-08 | 588578 | 0.0109 | 0.0139 | 0.4295 |
| Type 2 diabetes | rs6515236 | 20 | *SLC22A3* | C | -0.05 | 0.009 | 3.34E-08 | 565024 | 0.0275 | 0.016 | 0.08533 |
| Type 2 diabetes | rs67232546 | 11 | *LOC105372562 (FOXA2)* | T | 0.06 | 0.01 | 4.66E-10 | 574334 | 0.012 | 0.0169 | 0.4757 |
| Type 2 diabetes | rs6785040 | 3 | *ETS1* | C | -0.063 | 0.011 | 1.26E-08 | 555452 | 0.0059 | 0.0195 | 0.7603 |
| Type 2 diabetes | rs6795735 | 3 | *ADAMTS9-AS2* | T | -0.056 | 0.007 | 1.63E-14 | 678868 | 0.0181 | 0.0138 | 0.1896 |
| Type 2 diabetes | rs6808574 | 3 | *LOC107986166 (LPP)* | T | -0.055 | 0.008 | 4.39E-13 | 637074 | 0.0135 | 0.0139 | 0.3291 |
| Type 2 diabetes | rs687621 | 9 | *ABO* | G | 0.043 | 0.008 | 1.35E-08 | 691353 | 0.0053 | 0.0144 | 0.7119 |
| Type 2 diabetes | rs6878122 | 5 | *ZBED3-AS1* | G | 0.056 | 0.008 | 1.19E-12 | 646764 | 0.0142 | 0.0149 | 0.3415 |
| Type 2 diabetes | rs6960043 | 7 | *TSPAN8* | T | -0.064 | 0.007 | 3.61E-19 | 696176 | 0.0253 | 0.0137 | 0.06372 |
| Type 2 diabetes | rs7120121 | 11 | *NRXN3* | G | -0.055 | 0.01 | 6.09E-08 | 573004 | 0.0171 | 0.0174 | 0.3239 |
| Type 2 diabetes | rs7144011 | 14 | *LOC101929457 (HMG20A)* | T | 0.048 | 0.009 | 1.64E-08 | 703916 | 0.0199 | 0.0169 | 0.2383 |
| Type 2 diabetes | rs7177055 | 15 | *FTO* | G | -0.065 | 0.008 | 2.75E-16 | 693423 | 0.0019 | 0.0149 | 0.8995 |
| Type 2 diabetes | rs7185735 | 16 | *LAMA1* | G | 0.106 | 0.007 | 1.59E-47 | 686116 | 0.0263 | 0.0139 | 0.05805 |
| Type 2 diabetes | rs7240767 | 18 | *TFAP2B* | C | 0.045 | 0.008 | 2.16E-08 | 564455 | 0.008 | 0.0141 | 0.5718 |
| Type 2 diabetes | rs72802358 | 16 | *ACSL1* | C | -0.117 | 0.013 | 1.97E-18 | 542367 | 0.0508 | 0.0222 | 0.02191 |
| Type 2 diabetes | rs72892910 | 6 | *ZMIZ1* | T | 0.065 | 0.01 | 6.43E-11 | 626255 | -0.015 | 0.018 | 0.4049 |
| Type 2 diabetes | rs735949 | 4 | *SPHKAP* | C | -0.071 | 0.011 | 1.95E-11 | 642921 | 0.0136 | 0.0197 | 0.4899 |
| Type 2 diabetes | rs753270 | 10 | *RBMS1* | T | -0.053 | 0.008 | 2.7E-11 | 577383 | 0.0058 | 0.0139 | 0.6738 |
| Type 2 diabetes | rs7561798 | 2 | *IGF2BP2* | G | 0.04 | 0.007 | 2.79E-08 | 676590 | -0.011 | 0.0142 | 0.4375 |
| Type 2 diabetes | rs7572970 | 2 | *SLC9B2* | A | -0.059 | 0.009 | 1.39E-11 | 576484 | 0.0021 | 0.0152 | 0.8885 |
| Type 2 diabetes | rs7607777 | 2 | *TMEM154* | T | -0.137 | 0.013 | 9.4E-28 | 591814 | 0.013 | 0.022 | 0.5557 |
| Type 2 diabetes | rs7651090 | 3 | *CTRB1* | G | 0.12 | 0.008 | 3.85E-57 | 704418 | 0.0079 | 0.0147 | 0.5902 |
| Type 2 diabetes | rs7674212 | 4 | *PAM* | T | -0.047 | 0.008 | 6.18E-10 | 644144 | 0.0077 | 0.014 | 0.5839 |
| Type 2 diabetes | rs7685296 | 4 | *CDKAL1* | T | -0.051 | 0.008 | 2.32E-10 | 663001 | 0.0197 | 0.0152 | 0.1951 |
| Type 2 diabetes | rs7729395 | 5 | *UBE3C* | T | 0.137 | 0.016 | 1.1E-17 | 707542 | 0.0773 | 0.0369 | 0.03616 |
| Type 2 diabetes | rs7756992 | 6 | *GCKR* | G | 0.13 | 0.008 | 6E-62 | 735424 | 0.0065 | 0.0153 | 0.672 |
| Type 2 diabetes | rs7786095 | 7 | *SGK223* | G | -0.074 | 0.013 | 9.64E-09 | 565417 | 0.0233 | 0.0238 | 0.329 |
| Type 2 diabetes | rs780094 | 2 | *TP53INP1* | T | -0.069 | 0.007 | 5.16E-21 | 673973 | 0.0115 | 0.0138 | 0.4042 |
| Type 2 diabetes | rs7845219 | 8 | *TCF7L2* | C | -0.042 | 0.007 | 4.55E-09 | 675866 | 0.0104 | 0.0135 | 0.4423 |
| Type 2 diabetes | rs7903146 | 10 | *HHEX* | T | 0.306 | 0.008 | <1E-300 | 713516 | 0.0076 | 0.0147 | 0.6083 |
| Type 2 diabetes | rs7929543 | 11 | *TYRL* | C | 0.083 | 0.014 | 2.2E-09 | 603123 | 0.0435 | 0.0269 | 0.1049 |
| Type 2 diabetes | rs7931302 | 11 | *ETS1* | C | 0.046 | 0.008 | 7.65E-09 | 683446 | 0.0222 | 0.0154 | 0.1494 |
| Type 2 diabetes | rs7955901 | 12 | *ZZEF1* | C | 0.044 | 0.007 | 7.16E-10 | 684515 | 0.0114 | 0.0136 | 0.4039 |
| Type 2 diabetes | rs8068804 | 17 | *GIPR* | A | 0.059 | 0.008 | 4.41E-14 | 656020 | 0.0059 | 0.0145 | 0.6827 |
| Type 2 diabetes | rs8108269 | 19 | *ZNF664-FAM101A* | G | 0.064 | 0.008 | 3.11E-16 | 694468 | 0.0178 | 0.015 | 0.2336 |
| Type 2 diabetes | rs8182584 | 19 | *JAZF1* | T | 0.041 | 0.008 | 5.14E-08 | 663650 | 0.02 | 0.0142 | 0.1601 |
| Type 2 diabetes | rs825476 | 12 | *RPS4XP9* | C | -0.052 | 0.007 | 6.81E-13 | 674837 | 0.0076 | 0.0137 | 0.5808 |
| Type 2 diabetes | rs849135 | 7 | *LOC107986598 (VEGFA)* | G | 0.1 | 0.007 | 1.04E-43 | 675570 | 0.0028 | 0.0137 | 0.8352 |
| Type 2 diabetes | rs853974 | 6 | *PITPNM2* | T | 0.06 | 0.009 | 7.86E-12 | 584249 | 0.0081 | 0.0155 | 0.6039 |
| Type 2 diabetes | rs9369425 | 6 | *DLEU1* | G | 0.055 | 0.009 | 1.13E-10 | 586509 | 0.0034 | 0.0149 | 0.8213 |
| Type 2 diabetes | rs940904 | 12 | *USP3* | G | -0.05 | 0.008 | 2.08E-09 | 666360 | 0.0036 | 0.0153 | 0.812 |
| Type 2 diabetes | rs963740 | 13 | *LOC107986143 (TSC22D2)* | T | -0.048 | 0.009 | 2.23E-08 | 570121 | 0.0219 | 0.0148 | 0.1381 |
| Type 2 diabetes | rs9844972 | 3 | *UBE2Z* | C | 0.096 | 0.015 | 1.03E-10 | 616392 | 0.0174 | 0.0288 | 0.5458 |
| Type 2 diabetes | rs9894239 | 17 | *OSBPL7* | T | -0.059 | 0.008 | 1.55E-13 | 571299 | 0.0111 | 0.0137 | 0.4177 |
| Type 2 diabetes | rs993380 | 4 | *SCD5* | A | 0.051 | 0.008 | 4.59E-10 | 599636 | 0.027 | 0.0144 | 0.05995 |
| Type 2 diabetes | rs9940149 | 16 | *FAM234A* | A | -0.058 | 0.01 | 9.29E-10 | 661567 | 0.0022 | 0.0178 | 0.9024 |
| 2h glucose | rs1019503 | 5 | ERAP2 | A | 0.0628 | 0.0109 | 8.87E-09 | 133,010 | 0.0104 | 0.0136 | 0.4445 |
| 2h glucose | rs12243326 | 19 | TCF7L2 | C | 0.016229 | 0.002786 | 5.83E-09 | 133,010 | 0.0139 | 0.0169 | 0.4111 |
| 2h glucose | rs2877716 | 3 | ADCY5 | T | 0.022057 | 0.002588 | 1.65E-17 | 133,010 | 0.0038 | 0.0164 | 0.8187 |
| 2h glucose | rs11717195 | 8 |  | C | 0.0985 | 0.0165 | 2.15E-09 | 133,010 | 0.0153 | 0.0226 | 0.4999 |
| 2h glucose | rs10423928 | 10 | GIPR | T | 0.018395 | 0.002408 | 2.31E-14 | 133,010 | 0.0094 | 0.0153 | 0.5368 |
| 2h glucose | rs6975024 | 3 | GCKR | C | 0.01911 | 0.002631 | 3.95E-13 | 133,010 | 0.0024 | 0.0159 | 0.8814 |
| 2h glucose | rs11782386 | 7 | PPP1R3B | C | 0.1026 | 0.0156 | 5.25E-11 | 133,010 | 0.0147 | 0.018 | 0.412 |
| Fasting glucose | rs11605924 | 12 | CRY2 | A | 0.013344 | 0.002308 | 7.57E-09 | 133,010 | 0.0229 | 0.0162 | 0.1581 |
| Fasting glucose | rs11607883 | 9 |  | T | 0.02384 | 0.002757 | 5.65E-18 | 133,010 | -0.007 | 0.019 | 0.7105 |
| Fasting glucose | rs10814916 | 9 | GLIS3 | C | 0.015819 | 0.002156 | 2.26E-13 | 133,010 | 0.0073 | 0.0137 | 0.5962 |
| Fasting glucose | rs2191349 | 11 | DGKB/TMEM195 | G | 0.077887 | 0.002484 | 1.88E-07 | 133,010 | 0.0061 | 0.0159 | 0.7002 |
| Fasting glucose | rs780094 | 10 | GCKR | G | 0.027421 | 0.003278 | 6.32E-17 | 133,010 | 0.0034 | 0.0209 | 0.8715 |
| Fasting glucose | rs4502156 | 10 | VPS13C/C2CD4A/B | C | 0.032448 | 0.003702 | 1.97E-18 | 133,010 | 0.0108 | 0.024 | 0.6517 |
| Fasting glucose | rs7034200 | 8 | GLIS3 | A | 0.028879 | 0.002277 | 7.80E-37 | 133,010 | -0.001 | 0.0147 | 0.9459 |
| Fasting glucose | rs340874 | 11 | PROX1 | G | 0.019205 | 0.002826 | 1.12E-11 | 133,010 | 0.0077 | 0.019 | 0.6856 |
| Fasting glucose | rs174576 | 11 | FADS1 | A | 0.020187 | 0.002257 | 3.93E-19 | 133,010 | 0.0061 | 0.0136 | 0.6544 |
| Fasting glucose | rs174550 | 11 | FADS1 | G | 0.021346 | 0.002115 | 6.32E-24 | 133,010 | 0.0107 | 0.0137 | 0.4321 |
| Fasting glucose | rs4869272 | 13 | PCSK1 | G | 0.019523 | 0.002441 | 1.33E-15 | 133,010 | 0.0088 | 0.0164 | 0.593 |
| Fasting glucose | rs4506565 | 3 | TCF7L2 | A | 0.023016 | 0.002612 | 1.30E-18 | 133,010 | 0.0053 | 0.0166 | 0.7501 |
| Fasting glucose | rs11715915 | 3 | AMT | C | 0.012043 | 0.002206 | 4.90E-08 | 133,010 | 0.02 | 0.0146 | 0.1702 |
| Fasting glucose | rs7651090 | 3 | IGF2BP2 | T | 0.026374 | 0.003067 | 8.56E-18 | 133,010 | 0.0037 | 0.0197 | 0.851 |
| Fasting glucose | rs7903146 | 9 | TCF7L2 | T | 0.043415 | 0.00655 | 3.51E-11 | 133,010 | 0.0825 | 0.0453 | 0.06856 |
| Fasting glucose | rs11558471 | 11 | GCK | T | 0.018853 | 0.002206 | 1.34E-17 | 133,010 | 4.00E-04 | 0.0145 | 0.9786 |
| Fasting glucose | rs6943153 | 11 | GRB10 | C | 0.019743 | 0.002238 | 1.18E-18 | 133,010 | -0.001 | 0.0144 | 0.9462 |
| Fasting glucose | rs560887 | 6 | G6PC2 | T | 0.01212 | 0.002324 | 1.88E-07 | 133,010 | 0.0061 | 0.0155 | 0.6956 |
| Fasting glucose | rs7708285 | 7 | ZBED3 | T | 0.029157 | 0.00213 | 2.71E-20 | 133,010 | 0.017 | 0.0137 | 0.2137 |
| Fasting glucose | rs7944584 | 19 | MADD | C | 0.014358 | 0.002344 | 9.26E-10 | 133,010 | 0.0154 | 0.0179 | 0.3896 |
| Fasting glucose | rs9368222 | 12 | CDKAL1 | G | 0.012382 | 0.002727 | 5.69E-06 | 133,010 | 0.0143 | 0.0172 | 0.4037 |
| Fasting glucose | rs17762454 | 1 | RREB1 | C | 0.013464 | 0.002152 | 4.08E-10 | 133,010 | 0.0081 | 0.0139 | 0.5578 |
| Fasting glucose | rs10830963 | 14 | MTNR1B | G | 0.0168 | 0.002613 | 1.32E-10 | 133,010 | 0.0242 | 0.017 | 0.1551 |
| Fasting glucose | rs3829109 | 9 | DNLZ | G | 0.017249 | 0.002673 | 1.13E-10 | 133,010 | 0.0048 | 0.016 | 0.7645 |
| Fasting glucose | rs10747083 | 15 | P2RX2 | T | 0.022435 | 0.002144 | 1.38E-25 | 133,010 | 0.0057 | 0.0138 | 0.6776 |
| Fasting glucose | rs11619319 | 10 | PDX1 | T | 0.020595 | 0.002302 | 3.95E-19 | 133,010 | 0.0028 | 0.0146 | 0.8462 |
| Fasting glucose | rs11708067 | 5 | ADCY5 | T | 0.017677 | 0.002201 | 1.02E-15 | 133,010 | -0.004 | 0.0146 | 0.7838 |
| Fasting glucose | rs3783347 | 2 | WARS | C | 0.071132 | 0.002496 | 3.93E-19 | 133,010 | 0.0241 | 0.015 | 0.108 |
| Fasting glucose | rs2657879 | 13 | GLS2 | G | 0.016697 | 0.002984 | 2.26E-08 | 133,010 | 0.0076 | 0.019 | 0.6877 |
| Fasting glucose | rs2302593 | 20 | GIPR | A | 0.015924 | 0.002819 | 1.66E-08 | 133,010 | 0.0433 | 0.0195 | 0.02599 |
| Fasting glucose | rs576674 | 20 | KL | G | 0.035313 | 0.005287 | 2.49E-11 | 133,010 | 0.0237 | 0.0355 | 0.5034 |
| Fasting glucose | rs10811661 | 7 | CDKN2B | T | 0.015358 | 0.002173 | 1.63E-12 | 133,010 | 0.0138 | 0.015 | 0.3551 |
| Fasting glucose | rs11603334 | 9 | ARAP1 | A | 0.013394 | 0.002273 | 3.90E-09 | 133,010 | 0.0075 | 0.0138 | 0.5861 |
| Fasting glucose | rs6072275 | 3 | TOP1 | G | 0.012838 | 0.002276 | 1.75E-08 | 133,010 | 0.0079 | 0.0147 | 0.5902 |
| Fasting glucose | rs11920090 | 5 | SLC2A2 | G | 0.011438 | 0.002501 | 4.89E-06 | 133,010 | -0.014 | 0.0151 | 0.3564 |
| Fasting glucose | rs10885122 | 2 | ADRA2A | C | 0.027386 | 0.002144 | 2.58E-37 | 133,010 | 0.0115 | 0.0138 | 0.4042 |
| Fasting glucose | rs983309 | 10 | PPP1R3B | T | 0.021965 | 0.002378 | 2.71E-20 | 133,010 | 0.0076 | 0.0147 | 0.6083 |
| Fasting glucose | rs11195502 | 11 | ADRA2A | T | 0.023313 | 0.002414 | 4.82E-22 | 133,010 | 0.0002 | 0.0152 | 0.9893 |
| Fasting glucose | rs6113722 | 6 | FOXA2 | A | 0.014251 | 0.002331 | 1.00E-09 | 133,010 | 0.0082 | 0.0153 | 0.5914 |
| Fasting glucose | rs16913693 | 8 | IKBKAP | T | 0.025609 | 0.003282 | 6.29E-15 | 133,010 | 0.0184 | 0.0221 | 0.4058 |
| Fasting insulin | rs780094 | 13 | SLC30A3 | T | 0.017449 | 0.002106 | 1.26E-16 | 133,010 | 0.0069 | 0.0142 | 0.6274 |
| Fasting insulin | rs2943645 | 2 | MIR5702 | A | 0.015 | 0.003123 | 7.40E-10 | 133,010 | 0.0251 | 0.0193 | 0.1928 |
| Fasting insulin | rs3822072 | 13 | FAM13A1 | T | 0.021165 | 0.003042 | 3.59E-12 | 133,010 | 0.0214 | 0.0211 | 0.311 |
| Fasting insulin | rs10195252 | 13 | GRB14 | T | 0.023843 | 0.003272 | 3.30E-13 | 133,010 | 0.0175 | 0.0235 | 0.4564 |
| Fasting insulin | rs974801 | 13 | TET2 | T | 0.019336 | 0.002147 | 2.26E-19 | 133,010 | 0.0293 | 0.0141 | 0.03743 |
| Fasting insulin | rs4846565 | 13 | SLC30A10 | A | 0.011633 | 0.002064 | 1.80E-08 | 133,010 | 0.0185 | 0.0142 | 0.1902 |
| Fasting insulin | rs4865796 | 13 | ARL15 | G | 0.014655 | 0.002272 | 1.15E-10 | 133,010 | 0.0296 | 0.0157 | 0.05863 |
| Fasting insulin | rs731839 | 13 | PEPD | G | 0.013206 | 0.002192 | 1.76E-09 | 133,010 | 0.0012 | 0.0146 | 0.9355 |
| Fasting insulin | rs459193 | 13 | ANKRD55 | A | 0.015358 | 0.002185 | 2.16E-12 | 133,010 | 0.0228 | 0.0147 | 0.1218 |
| Fasting insulin | rs6912327 | 0 | PPARD | A | 0.013819 | 0.002184 | 2.58E-10 | 133,010 | 0.0329 | 0.0187 | 0.07852 |
| Fasting insulin | rs860598 | 13 | IGF1 | T | 0.016472 | 0.002944 | 2.26E-08 | 133,010 | 0.0358 | 0.0163 | 0.02838 |
| Fasting insulin | rs6822892 | 13 | PDGFC | G | 0.014846 | 0.002149 | 5.13E-12 | 133,010 | 0.0235 | 0.015 | 0.1173 |
| Fasting insulin | rs1530559 | 13 | YSK4 | C | 0.027386 | 0.002144 | 2.58E-37 | 133,010 | 0.0115 | 0.0138 | 0.4042 |
| Fasting insulin | rs17036328 | 13 | PPARG | A | 0.004818 | 0.002773 | 4.89E-06 | 133,010 | 0.0213 | 0.0182 | 0.2419 |
| Fasting insulin | rs2126259 | 13 | PPP1R3B | G | 0.013936 | 0.002099 | 3.27E-11 | 133,010 | 0.0015 | 0.0144 | 0.9193 |
| Fasting proinsulin | rs4790333 | 11 | SGSM2 | G | 0.0775 | 0.0064 | 1.10E-88 | 16,378 | -0.004 | 0.0156 | 0.7982 |
| Fasting proinsulin | rs4502156 | 11 | VPS13C/C2CD4A/B | T | 0.0246 | 0.0049 | 6.90E-12 | 16,378 | -0.029 | 0.017 | 0.08749 |
| Fasting proinsulin | rs1549318 | 8 | RPL29P30 | A | 0.0273 | 0.0048 | 3.10E-18 | 16,378 | -0.001 | 0.0147 | 0.9459 |
| Fasting proinsulin | rs9727115 | 11 | SNX7 | A | 0.0938 | 0.0052 | 3.20E-102 | 16,378 | 0.0077 | 0.019 | 0.6856 |
| Fasting proinsulin | rs7903146 | 15 | TCF7L2 | T | 0.018 | 0.005 | 2.40E-10 | 16,378 | 7.00E-04 | 0.0139 | 0.9571 |
| Fasting proinsulin | rs11558471 | 15 | SLC30A8 | T | 0.026 | 0.0043 | 3.50E-20 | 16,378 | 0.0057 | 0.0138 | 0.6776 |
| Fasting proinsulin | rs6235 | 17 | PCSK1 | T | 0.0179 | 0.0042 | 3.00E-09 | 16,378 | 0.0023 | 0.0138 | 0.8668 |
| Fasting proinsulin | rs10501320 | 5 | MADD | G | 0.0438 | 0.0049 | 9.80E-27 | 16,378 | 0.018 | 0.0154 | 0.2409 |
| Fasting proinsulin | rs10838687 | 10 | MADD | T | 0.0303 | 0.0063 | 2.30E-20 | 16,378 | 0.0076 | 0.0147 | 0.6083 |
| Fasting proinsulin | rs11603334 | 1 | ARAP1 | G | 0.0134 | 0.0045 | 2.40E-07 | 16,378 | 0.0025 | 0.0143 | 0.8625 |
| HbA1C | rs855791 | 17 | TMPRSS6 | T | 0.035 | 0.003 | 1.57E-26 | 46,368 | 0.0062 | 0.0144 | 0.6643 |
| HbA1C | rs552976 | 11 | ABCB11 | T | 0.028 | 0.004 | 3.96E-11 | 46,368 | 0.0105 | 0.015 | 0.4831 |
| HbA1C | rs1046896 | 10 | FN3K | C | 0.089 | 0.004 | 3.11E-54 | 46,368 | 0.002 | 0.0209 | 0.9227 |
| HbA1C | rs1387153 | 7 | MTNR1B | T | 0.038 | 0.004 | 1.45E-20 | 46,368 | 0.0133 | 0.0179 | 0.4567 |
| HbA1C | rs2779116 | 6 | SPTA1 | G | 0.063 | 0.007 | 2.59E-20 | 46,368 | 0.0229 | 0.0303 | 0.4487 |
| HbA1C | rs4737009 | 1 | ANK1 | T | 0.024 | 0.004 | 2.75E-09 | 46,368 | 0.0044 | 0.0153 | 0.7708 |
| HbA1C | rs1799884 | 8 | GCK | A | 0.027 | 0.004 | 6.11E-12 | 46,368 | 0.028 | 0.0166 | 0.09196 |
| HbA1C | rs7998202 | 2 | ATP11AUN | G | 0.047 | 0.003 | 8.16E-18 | 46,368 | 0.022 | 0.0143 | 0.1225 |
| HbA1C | rs16926246 | 8 | HK1 | T | 0.058 | 0.011 | 1.18E-08 | 46,368 | 0.0065 | 0.0402 | 0.8724 |
| HbA1C | rs1800562 | 13 | HFE | G | 0.031 | 0.005 | 5.24E-09 | 46,368 | 0.0119 | 0.02 | 0.5515 |
| HbA1C | rs6474359 | 22 | ANK1 | A | 0.027 | 0.004 | 2.74E-14 | 46,368 | 0.0135 | 0.0137 | 0.3252 |

SNPs: single nucleotide polymorphisms; Chr, chromosome; EA, effect allele; SE: Standard Error; NGWAS: Number of Genome wide association studies; T2D=Type 2 diabetes mellitus; HbA1C = hemoglobin A1c. The heterogeneity of these SNPs for T2D was PHEIDI > 7.8 × 10−4. The I2 (I squared) value for the T2D instrumental variables in European was 17.3% in Fixed model.

**Table S2.** Summary statistics for the genetic variants associated with T2D and related traits investigated for an association with ALS in East Asian ancestry.

| **Risk factors** | **SNP** | **Chr** | **Nearest gene** | **EA** | **Risk Factor** | | | **NGWAS** | **ALS** | | |
| --- | --- | --- | --- | --- | --- | --- | --- | --- | --- | --- | --- |
|  |  |  |  |  | **Beta** | **SE** | **P value** |  | **Beta** | **SE** | **P value** |
| T2D | rs10814916 | 9 | GLIS3 | C | 0.1044 | 0.0178 | 6.01E-12 | 16005 | -0.0046 | 0.0483 | 0.9236 |
| T2D | rs10886471 | 10 | GRK5 | C | 0.1133 | 0.0203 | 7.10E-09 | 16005 | 0.0703 | 0.0603 | 0.243 |
| T2D | rs11257655 | 10 | CDC123 | T | 0.1398 | 0.0254 | 6.56E-09 | 16005 | -0.0286 | 0.0489 | 0.5618 |
| T2D | rs2206734 | 6 | CDKAL1 | A | 0.3148 | 0.0660 | 7.17E-11 | 1999 | -0.0361 | 0.0496 | 0.4669 |
| T2D | rs2299620 | 11 | KCNQ1 | G | 0.3148 | 0.0711 | 4.24E-10 | 16005 | -0.0862 | 0.0499 | 0.08287 |
| T2D | rs2383208 | 9 | CDKN2B | A | 0.1989 | 0.0279 | 3.38E-17 | 16005 | -0.0129 | 0.0489 | 0.7983 |
| T2D | rs4430796 | 17 | HNF1B | G | 0.1740 | 0.0305 | 1.52E-11 | 16005 | -0.1128 | 0.0542 | 0.03725 |
| T2D | rs7403531 | 15 | RASGRP1 | T | 0.0953 | 0.0178 | 3.90E-09 | 16005 | -0.0036 | 0.0504 | 0.9422 |
| Glucose | rs10811661 | 9 | CDKN2A/B | T | 0.0620 | 0.0090 | 8.66E-12 | 24740 | 0.0185 | 0.0487 | 0.7036 |
| Glucose | rs10815355 | 9 | KANK1 | T | 0.0450 | 0.0070 | 1.26E-09 | 46085 | 0.0497 | 0.0576 | 0.3869 |
| Glucose | rs13387347 | 2 | G6PC2-ABCC11 | C | 0.1140 | 0.0090 | 2.35E-36 | 24740 | 0.0257 | 0.0483 | 0.5992 |
| Glucose | rs1974620 | 7 | TMEM195 | T | 0.0630 | 0.0090 | 2.79E-11 | 24740 | -0.0507 | 0.0496 | 0.307 |
| Glucose | rs2018860 | 15 | IGF1R | A | 0.0310 | 0.0060 | 2.99E-08 | 46085 | 0.0227 | 0.0477 | 0.6371 |
| Glucose | rs3802177 | 8 | SLC30A8 | G | 0.0630 | 0.0090 | 5.23E-12 | 24740 | 0.0213 | 0.0495 | 0.6665 |
| Glucose | rs3847554 | 11 | MTNR1B | T | 0.0590 | 0.0090 | 2.20E-11 | 24740 | -0.0078 | 0.0483 | 0.8717 |
| Glucose | rs4237150 | 9 | GLIS3 | C | 0.0530 | 0.0090 | 4.31E-09 | 24740 | -0.0120 | 0.0489 | 0.8073 |
| Glucose | rs6048216 | 20 | FOXA2 | T | 0.0950 | 0.0130 | 1.91E-12 | 24740 | 0.0658 | 0.0739 | 0.3726 |
| Glucose | rs730497 | 7 | GCK | A | 0.1210 | 0.0110 | 7.72E-27 | 24740 | 0.1124 | 0.0594 | 0.05871 |
| Glucose | rs733331 | 2 | PDK1-RAPGEF4 | A | 0.0360 | 0.0060 | 6.98E-11 | 46085 | 0.0495 | 0.0488 | 0.3105 |
| Glucose | rs780094 | 2 | GCKR | C | 0.0520 | 0.0090 | 3.58E-09 | 24740 | -0.0036 | 0.0484 | 0.9412 |
| Glucose | rs895636 | 2 | SIX2-SIX3 | T | 0.0690 | 0.0100 | 2.53E-13 | 24740 | 0.0431 | 0.0499 | 0.3839 |
| Glucose | rs9356744 | 6 | CDKAL1 | C | 0.0570 | 0.0090 | 9.24E-10 | 24740 | -0.0123 | 0.0496 | 0.8041 |
| Glycated Hemoglobin | rs1046875 | 17 | FN3K | A | 0.0800 | 0.0100 | 1.60E-14 | 20871 | -0.0607 | 0.0487 | 0.2129 |
| Glycated Hemoglobin | rs11667918 | 19 | MYO9B | C | 0.0600 | 0.0100 | 1.90E-10 | 20835 | -0.0592 | 0.0500 | 0.2394 |
| Glycated Hemoglobin | rs1467311 | 9 | 9q31.2 | G | 0.0400 | 0.0100 | 2.90E-08 | 20845 | -0.0177 | 0.0577 | 0.7593 |
| Glycated Hemoglobin | rs174570 | 11 | FADS2 | C | 0.0400 | 0.0100 | 5.40E-07 | 20639 | -0.0402 | 0.0506 | 0.4319 |
| Glycated Hemoglobin | rs1799884 | 7 | GCK | T | 0.1200 | 0.0100 | 1.50E-22 | 20874 | 0.1098 | 0.0594 | 0.06431 |
| Glycated Hemoglobin | rs3755157 | 2 | G6PC2/ABCB11 | T | 0.0700 | 0.0100 | 2.80E-11 | 20630 | 0.0658 | 0.0515 | 0.2022 |
| Glycated Hemoglobin | rs540078 | 11 | PSMD13 | T | 0.0300 | 0.0100 | 6.20E-06 | 20865 | 0.0218 | 0.0485 | 0.6501 |
| Glycated Hemoglobin | rs6684514 | 1 | TMEM79 | G | 0.0900 | 0.0100 | 1.10E-15 | 20831 | -0.0050 | 0.0560 | 0.9265 |
| Glycated Hemoglobin | rs7772603 | 6 | CDKAL1 | C | 0.0600 | 0.0100 | 3.50E-08 | 19156 | 0.0208 | 0.0497 | 0.6706 |
| Glycated Hemoglobin | rs9399137 | 6 | HBS1L/MYB | T | 0.0700 | 0.0100 | 1.90E-08 | 20535 | -0.0797 | 0.0518 | 0.1244 |
| Glycated Hemoglobin | rs9933309 | 16 | CYBA | C | 0.0700 | 0.0100 | 3.30E-08 | 11015 | -0.1441 | 0.0505 | 0.004311 |
| BMI | rs10938397 | 4 | GNPDA2 | G | 3.7200 | 0.8500 | 9.69 ×E-8 | 65406 | 0.0545 | 0.0519 | 0.2968 |
| BMI | rs11671664 | 19 | GIPR-QPCTL | G | 4.2200 | 0.7600 | 5.93 × 10^−14^ | 83048 | -0.0139 | 0.0487 | 0.7786 |
| BMI | rs12597579 | 16 | GP2 | C | 4.0900 | 0.9600 | 1.02 × 10^−8^ | 83048 | -0.0971 | 0.0537 | 0.07078 |
| BMI | rs17817449 | 16 | FTO | G | 7.9200 | 1.0600 | 4.60 × 10^−27^ | 65406 | -0.0375 | 0.0748 | 0.6159 |
| BMI | rs261967 | 5 | PCSK1 | C | 3.7700 | 0.7700 | 5.13 × 10^−9^ | 83048 | -0.0966 | 0.0490 | 0.0486 |
| BMI | rs4715210 | 6 | TFAP2B | T | 3.0500 | 0.9100 | 1.61 × 10^−7^ | 65406 | -0.0667 | 0.0655 | 0.3083 |
| BMI | rs4776970 | 15 | MAP2K5 | A | 2.5500 | 0.9000 | 2.33 × 10^−9^ | 83048 | 0.0545 | 0.0556 | 0.325 |
| BMI | rs574367 | 1 | SEC16B | T | 5.9300 | 0.9200 | 9.47 × 10^−20^ | 65406 | 0.0373 | 0.0604 | 0.5411 |
| BMI | rs6265 | 11 | BDNF | C | 4.9700 | 0.8300 | 3.56 × 10^−13^ | 65406 | 0.0499 | 0.0485 | 0.3034 |
| BMI | rs652722 | 11 | PAX6 | C | 2.7500 | 0.7700 | 7.65 × 10^−8^ | 83048 | 0.0456 | 0.0509 | 0.3698 |
| BMI | rs6545814 | 2 | ADCY3-DNAJC27 | G | 3.2600 | 0.7600 | 1.35 × 10^−13^ | 83048 | 0.0411 | 0.0490 | 0.3978 |
| BMI | rs6567160 | 18 | MC4R | C | 5.5100 | 0.9300 | 2.76 × 10^−15^ | 65406 | 0.0315 | 0.0583 | 0.5852 |
| WCadjBMI | rs2057291 | 20 | GNAS | G | 2.5200 | 0.4600 | 4.02E-08 | 38613 | -0.0060 | 0.0542 | 0.9083 |
| WCadjBMI | rs3791679 | 2 | EFEMP1 | A | 2.8700 | 0.3800 | 4.86E-14 | 64454 | 0.0334 | 0.0593 | 0.5718 |
| WCadjBMI | rs3809128 | 12 | CNPY2 | C | 3.6900 | 0.6300 | 3.74E-09 | 30368 | -0.0257 | 0.0587 | 0.6624 |
| WCadjBMI | rs8030379 | 15 | ADAMTSL3 | A | 2.4600 | 0.4100 | 1.62E-09 | 50668 | 0.0378 | 0.0582 | 0.5162 |

SNPs: single nucleotide polymorphisms; Chr, chromosome; EA, effect allele; SE: Standard Error; NGWAS: Number of Genome wide association studies; T2D=Type 2 diabetes mellitus; BMI: Body mass index. The heterogeneity of these SNPs for T2D was P=3.33 × 10−4. The I2 (I squared) value for the T2D instrumental variables in East Asian populations was 0.0% in Fixed model.

**Table S3.** Results of four MR methods of MR analysis for T2D and related traits in European ancestry.

| **Risk factors** | **Simple median** | | | | **Weighted median** | | | | | **IVW** | | | | | **MREgger** | | | | | | | | |
| --- | --- | --- | --- | --- | --- | --- | --- | --- | --- | --- | --- | --- | --- | --- | --- | --- | --- | --- | --- | --- | --- | --- | --- |
|  | **Beta** | **SE** | | **P-value** | **Beta** | | **SE** | **P-value** | | **Beta** | | **SE** | | **P-value** | **Beta** | | **SE** | | **P-value** | | **(intercept)** | **P-value** | |
| T2D | -0.062 | 0.029 | | 0.035 | -0.027 | | 0.037 | 0.457 | | -0.044 0.020 | | | | 0.030 | -0.065 0.047 | | | | 0.162 | | 0.002±0.003 | 0.615 | |
| 2h glucose | 0.166 | 0.216 | | 0.444 | -0.139 | | 0.141 | 0.322 | | -0.031 | | 0.113 | | 0.785 | -0.203 | | 0.189 | | 0.282 | | 0.012±0.010 | 0.255 | |
| Fasting glucose | -0.013 | 0.155 | | 0.933 | 0.096 | | 0.142 | 0.498 | | 0.136 | | 0.097 | | 0.162 | 0.175 | | 0.186 | | 0.347 | | -0.001±0.005 | 0.809 | |
| Fasting insulin | -0.395 | 0.409 | | 0.333 | -0.403 | | 0.379 | 0.288 | | -0.189 | | 0.339 | | 0.577 | 0.48 | | 1.177 | | 0.684 | | -0.012±0.020 | 0.552 | |
| Fasting proinsulin | -0.044 | 0.185 | | 0.811 | -0.061 | | 0.141 | 0.665 | | -0.038 | | 0.116 | | 0.744 | -0.021 | | 0.205 | | 0.918 | | -0.001±0.008 | 0.922 | |
| HbA1C | 0.112 | 0.19 | 0.555 | | 0.093 | 0.167 | | | 0.576 | | 0.123 | 0.128 | 0.337 | | | 0.168 | | 0.32 | 0.6 | -0.002±0.013 | | | 0.879 |

HbA1C: hemoglobin A1c; SE: Standard Error; IVW: Inverse‐Variance Weighted Method; MR: Mendelian randomization; BMI: body mass index; WHRadjBMI: waist-to-hip ratio adjusted for BMI; MREgger: Mendelian randomization-Egger.

**Table S4.** Results of four MR methods of MR analysis for T2D and related traits in East Asian

ancestry.

| **Risk factors** | **Simple median** | | | **Weighted median** | | | **IVW** | | | **MREgger** | | | | |
| --- | --- | --- | --- | --- | --- | --- | --- | --- | --- | --- | --- | --- | --- | --- |
|  | **Beta** | **SE** | **P-value** | **Beta** | **SE** | **P-value** | **Beta** | **SE** | **P-value** | **Beta** | **SE** | **P-value** | **(intercept)** | **P-value** |
| T2D | -0.09 | 0.128 | 0.483 | -0.17 | 0.115 | 0.138 | -0.182 | 0.089 | 0.040 | -0.303 | 0.213 | 0.155 | 0.027±0.043 | 0.533 |
| Glucose | 0.318 | 0.276 | 0.248 | 0.284 | 0.271 | 0.295 | 0.325 | 0.201 | 0.105 | 0.559 | 0.556 | 0.314 | -0.017±0.038 | 0.652 |
| Glycated Hemoglobin | -0.441 | 0.412 | 0.284 | -0.208 | 0.356 | 0.558 | -0.218 | 0.311 | 0.482 | 0.58 | 0.941 | 0.538 | -0.058±0.065 | 0.368 |
| BMI | 0.006 | 0.005 | 0.225 | 0.006 | 0.005 | 0.242 | 0 | 0.004 | 0.93 | -0.003 | 0.014 | 0.836 | 0.015±0.062 | 0.809 |
| WCadjBMI | 0.005 | 0.012 | 0.689 | -0.002 | 0.012 | 0.879 | 0.002 | 0.01 | 0.827 | -0.035 | 0.059 | 0.557 | 0.109±0.172 | 0.527 |

SE: Standard Error; IVW: Inverse‐Variance Weighted Method; MR: Mendelian randomization; BMI: body mass index; WHRadjBMI: waist-to-hip ratio adjusted for BMI; MREgger: Mendelian Randomization-Egger.

**Table S5.** Genetic variants of T2D in European and East Asian ancestry that were associated with other traits.

| **Risk factors** | **SNP** | **Chr** |  | **Gene** | **EA** | **other traits** | **P-values** | |
| --- | --- | --- | --- | --- | --- | --- | --- | --- |
| **European ancestry** | | | | | | | |  |
| Type 2 diabetes | rs10401969 | 19 |  | *BCL2L11* | C | alcoholic liver cirrhosis | 8 x 10-10 | |
|  |  |  |  |  |  | total cholesterol measurement | 3 x 10-38 | |
| Type 2 diabetes | rs1042725 | 12 |  | *SUGP1* | T | [body height](https://www.ebi.ac.uk/gwas/efotraits/EFO_0004339) | 3 x 10-18 | |
|  |  |  |  |  |  | [infant head circumference](https://www.ebi.ac.uk/gwas/efotraits/EFO_0004577) | 3 x 10-10 | |
| Type 2 diabetes | rs10811661 | 9 |  | *CDKN2B-AS1* | C | BMI | 5 x 10-13 | |
|  |  |  |  |  |  | [fasting blood glucose measurement](https://www.ebi.ac.uk/gwas/efotraits/EFO_0004465) | 9 x 10-12 | |
| Type 2 diabetes | rs11107116 | 12 |  | *NUDT6* | T | [body height](https://www.ebi.ac.uk/gwas/efotraits/EFO_0004339) | 1 x 10-34 | |
| Type 2 diabetes | rs12970134 | 18 |  | *RAI1* | A | waist circumference | 2 x 10-9 | |
|  |  |  |  |  |  | [body weight](https://www.ebi.ac.uk/gwas/efotraits/EFO_0004338) | 5 x 10-13 | |
| Type 2 diabetes | rs13389219 | 2 |  | *CTTNBP2* | T | [waist-hip ratio](https://www.ebi.ac.uk/gwas/efotraits/EFO_0004343) | 3 x 10-8 | |
| Type 2 diabetes | rs1899951 | 3 |  | *UBAP2* | T | body mass index | 6 x 10-9 | |
| Type 2 diabetes | rs2307111 | 5 |  | *ZNF34* | C | HDL cholesterol | 3 x 10-16 | |
|  |  |  |  |  |  | [obesity](https://www.ebi.ac.uk/gwas/efotraits/EFO_0001073) | 3 x 10-12 | |
| Type 2 diabetes | rs2867125 | 2 |  | *LOC102723886 (LYPLAL1)* | T | physical activity measurement, | 1 x 10-49 | |
|  |  |  |  |  |  | body mass index | 1 x 10-72 | |
| Type 2 diabetes | rs2925979 | 16 |  | *TMEM18* | T | HDL cholesterol | 6 x 10-27 | |
|  |  |  |  |  |  | BMI-adjusted waist-hip ratio | 7 x 10-13 | |
| Type 2 diabetes | rs340874 | 1 |  | *MIR5702* | T | Fasting blood glucose | 7 x 10-12 | |
| Type 2 diabetes | rs459193 | 5 |  | *C5orf67* | A | triglyceride measurement | 9 x 10-29 | |
|  |  |  |  |  |  | HDL cholesterol | 2 x 10-23 | |
|  |  |  |  |  |  | smoking behavior, | 4 x 10-20 | |
| Type 2 diabetes | rs6795735 | 3 |  | *ADAMTS9-AS2* | T | Waist-hip ratio | 1 x 10-13 | |
|  |  |  |  |  |  | Diastolic blood pressure | 1 x 10-11 | |
| Type 2 diabetes | rs687621 | 9 |  | *ABO* | G | blood protein measurement | 4 x 10-17 | |
| Type 2 diabetes | rs7144011 | 14 |  | *LOC101929457 (HMG20A)* | T | waist circumference | 9 x 10-16 | |
| Type 2 diabetes | rs7185735 | 16 |  | *LAMA1* | G | obesity | 1 x 10-79 | |
| Type 2 diabetes | rs7756992 | 6 |  | *GCKR* | G | a1c measurement | 3 x 10-12 | |
| Type 2 diabetes | rs780094 | 2 |  | *TP53INP1* | T | fasting blood glucose measurement | 3 x 10-24 | |
| **East Asian ancestry** |  |  |  |  |  |  |  | |
| Type 2 diabetes | rs2206734 | 6 |  | *CDKAL1* | A | body mass index | 1 x 10-11 | |

SNPs: single nucleotide polymorphisms; Chr, chromosome; EA, effect allele;

SE: Standard Error; NGWAS: Number of Genome wide association studies; BMI: Body mass index.
